# Standardized Quorum Sensing Tools for Gram-Negative Bacteria

**DOI:** 10.1021/acssynbio.5c00036

**Published:** 2025-06-06

**Authors:** Paula Múgica-Galán, Jesús Miró-Bueno, Ángeles Hueso-Gil, Pablo Japón, Ángel Goñi-Moreno

**Affiliations:** † Systems Biology Department, Centro Nacional de Biotecnologia, CSIC, Darwin 3, 28049 Madrid, Spain; ‡ Universidad Politécnica de Madrid, Madrid 28223, Spain

**Keywords:** quorum sensing, standards, distributed computation, genetic tools, cell−cell communication, bacteria

## Abstract

Engineering synthetic consortia to perform distributed functions requires robust quorum sensing (QS) systems to facilitate communication between cells. However, the current QS toolbox lacks standardized implementations, which are particularly valuable for use in bacteria beyond the model species Escherichia coli. We developed a set of three QS systems encompassing both sender and receiver modules, constructed using backbones from the SEVA (Standard European Vector Architecture) plasmid collection. This increases versatility, allowing plasmid features like the origin of replication or antibiotic marker to be easily swapped. The systems were characterized using the synthetic biology chassis Pseudomonas putida. We first tested individual modules, then combined sender and receiver modules in the same host, and finally assessed the performance across separate cells to evaluate consortia dynamics. Alongside the QS set, we provide mathematical models and rate parameters to support the design efforts. Together, these tools advance the engineering of robust and predictable multicellular functions.

## Graphical abstract

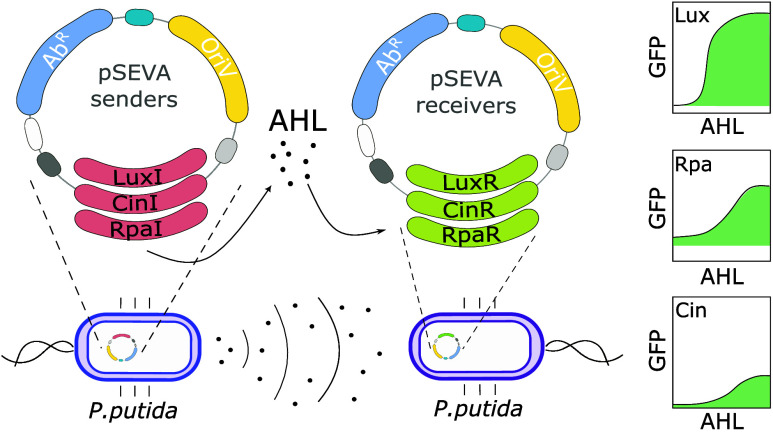

## Introduction

The design and implementation of multicellular functions in bacterial consortia have been a key goal of synthetic biology for years.^1^ This effort is driven by the potential to harness the capabilities of natural populations, where interactions among cells can give rise to novel functions, functions that are difficult or even impossible to achieve at the single-cell level.

Central to this approach is the establishment of communication channels that enable cells to send and receive molecular information. Among the various strategies for implementing these channels, such as using phages^2^ or bacterial conjugation^3^ to transfer genetic elements, quorum sensing (QS) systems have been most widely utilized for synthetic biology and circuit engineering purposes.^4–7^ A QS sender module produces acylhomoserine lactones (AHLs), small signaling molecules that diffuse through the cell membrane and travel to neighboring cells. These molecules are detected by a QS receiver module in the recipient cells, enabling communication between them. These on/off molecular communication systems have been extensively employed in Escherichia coli for applications, including gene expression control,^8^ pattern formation,^9^ metabolic engineering,^10^ and computation.^11^ However, their use in Gram-negative bacteria beyond E. coli remains relatively unexplored.

To address this gap, we standardized QS mechanisms by building constructs compatible with the SEVA (Standard European Vector Architecture) format,^12^ which is designed for and used in different Gram-negative bacterial strains such as Pseudomonas putida,^13^ Pseudomonas protegens,^14^ E. coli,^15^ or Pseudomonas aeruginosa.^16^ Furthermore, we have characterized their performance in the soil bacterium P. putida, an increasingly used host, because of its robustness, versatile metabolism, high reducing power, and tolerance to stresses.^17^

## Results and Discussion

### Standardization of QS Systems

The QS sender modules were designed by using the XylS-*Pm* inducible promoter system to regulate the expression of synthase enzymes responsible for producing AHL signaling molecules. These modules include the lux system from Vibrio fischeri, the rpa system from Rhodopseudomonas palustris, and the cin system from Rhizobium leguminosarum. For the QS receiver modules, we employed those previously characterized in E. coli by Kylilis et al.^18^ (Figure 1). These modules use the constitutive promoter *BBa*_J_23101 to drive the expression of the transcription factor that binds to the AHL signaling molecule. This complex then activates the QS promoter, triggering the expression of the green fluorescent protein (GFP) encoded downstream.

**Figure 1:**
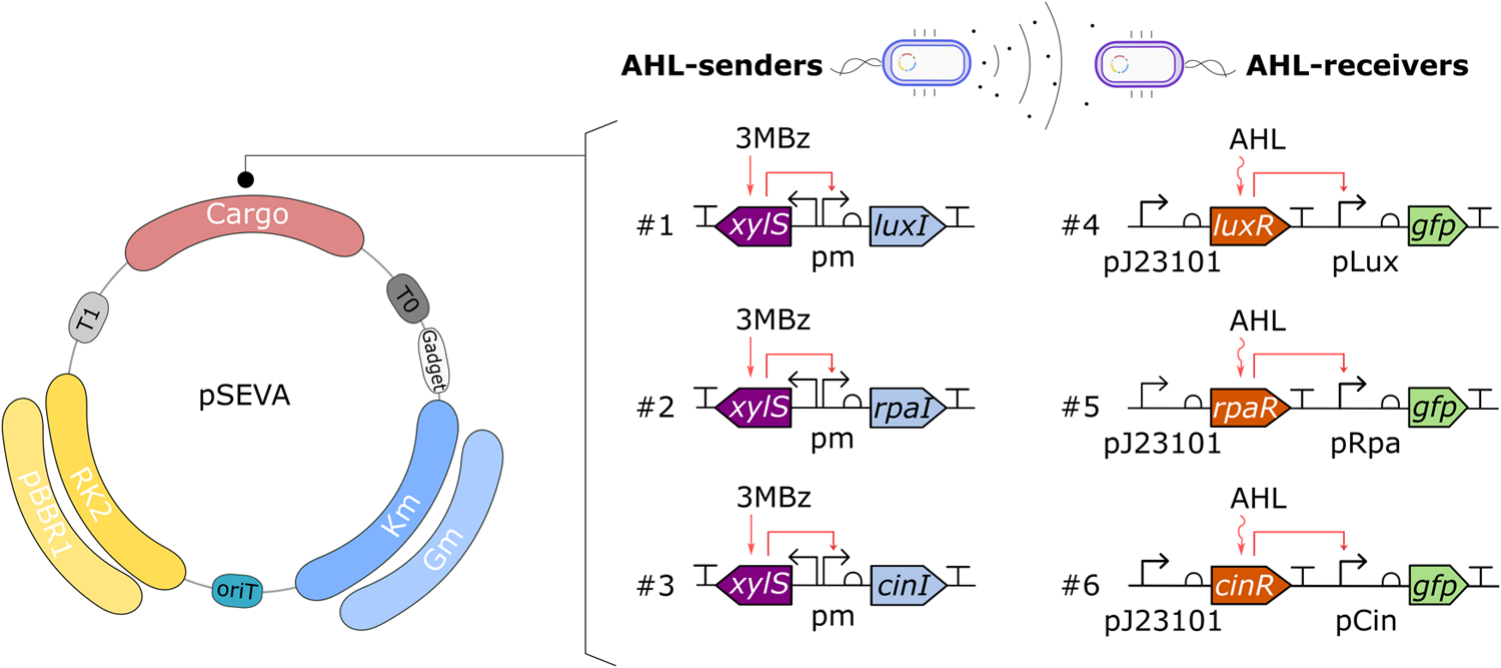
Plasmid map and genetic architecture of the standardized QS systems. Each number corresponds to a different constructed plasmid, the genes colored in blue are the AHL-synthases (sender modules), whose expression is induced by 3MBz, and the ones in orange are the transcription factor of the QS systems (receiver modules).

We placed the modules on vectors from the SEVA collection,^12^ enabling modular exchange of origins of replication and selection markers (Figure 1). Sender modules were cloned onto a medium copy-number plasmid (origin pBBR1) to ensure that enough AHL molecules were produced, while receiver modules were placed on a low copy-number plasmid (origin RK2) to balance transcription factor expression and reduce the burden on the host cell. We tested these constructs in the soil bacterium P. putida in three configurations: [i] Receiver only: Chemically induced with AHL molecules to validate their response. [ii] Sender and receiver in the same cell: Both modules were coexpressed within a single strain to test their functionality. [iii] Two-strain interaction: Senders and receivers were separated into two distinct strains to evaluate their performance in a mixed population.

### Performance of Receiver Modules

P. putida cells were transformed with the corresponding plasmids and cultured in the presence of their cognate synthetic AHL, and the GFP signal was measured using a plate reader. As shown in Figure 2A, the three receivers respond to increasing concentrations of AHLs in the host P. putida KT2440. However, the response curves differ between them. For instance, the cin system has the narrowest operational range (the range of ligand concentrations that induce a response), whereas rpa and lux exhibit responses over a wider range of AHL concentrations. In terms of fold change (Figure 2C) (the ratio between maximum fluorescence and basal expression), lux and cin show the highest values, while rpa achieves only a 2-fold induction. This limited fold change is partly due to high promoter leakiness but may also result from system saturation. This issue could be addressed by optimizing the promoter and RBS sequences using strategies such as directed evolution^19,20^ or tools like the Salis calculator,^21,22^ which allows the design of new sequences with predicted strength.

**Figure 2:**
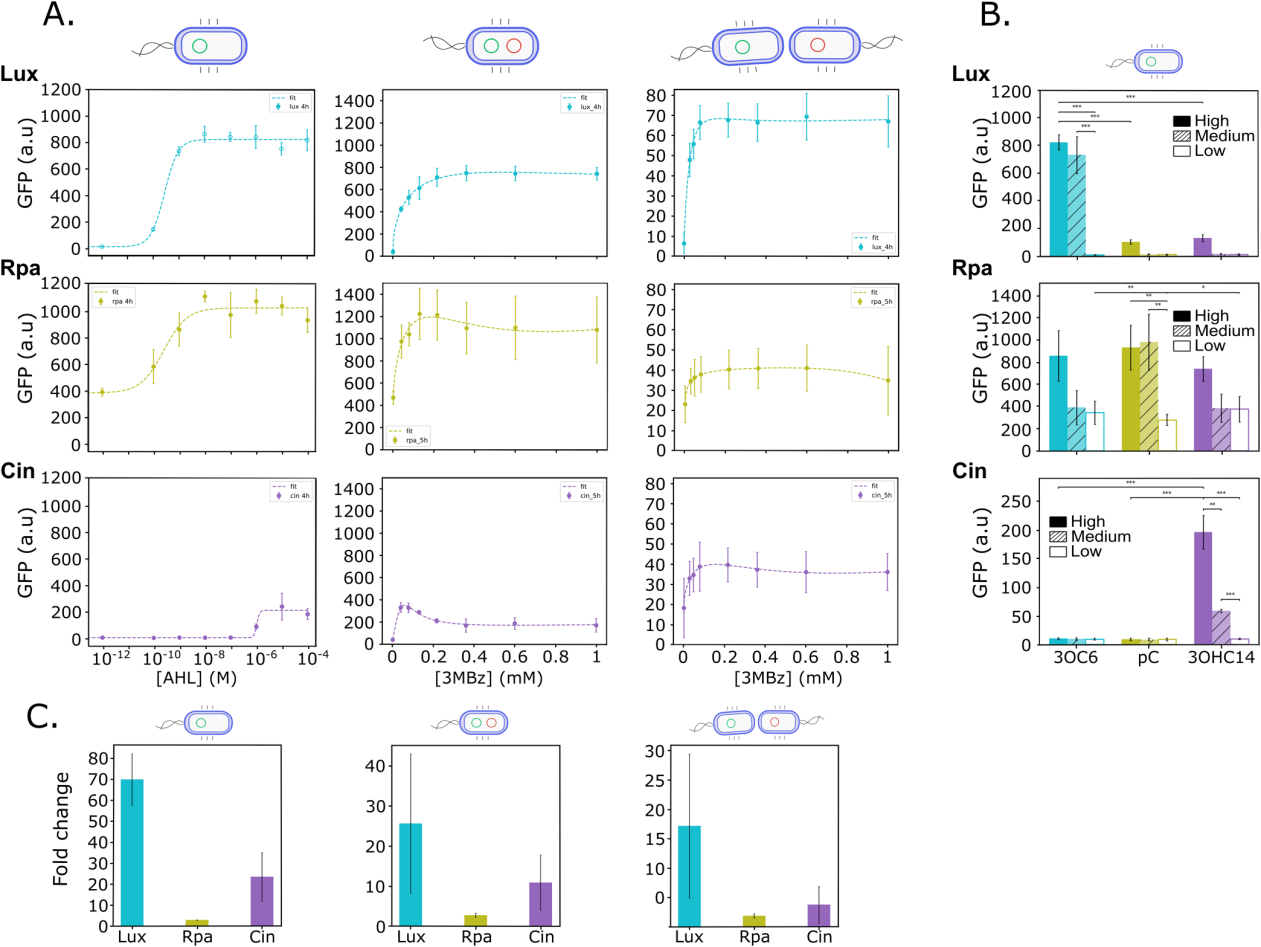
Characterization of the standardized QS systems in P. putida. (A) Response curves of the systems in the three different configurations: receiver modules, single strain, or two strains. The input is either AHL for receiver modules or 3MBz for single and two-strain experiments, and the output is GFP fluorescence in arbitrary units (au). Dots show experimental data in terms of the mean of three biological replicates, and error bars represent standard deviation. The dashed lines connecting the dots show the fitted model. (B) Bar charts representing the induction of the receivers with every AHL molecule. The concentrations of the noncognate were 10^–10^ M (low), 10^–6^ M (medium), and 10^–4^ M (high). Cognate molecules were used at 0 M (low), 10^–6^ M (medium), and 10^–4^ M (high). *t* test statistical analyses were performed to compare the GFP fluorescence output as a result of the different cognate AHL concentrations on each system. Additionally, cross-talk was also analyzed by measuring the fluorescence output of receivers when induced by their noncognate molecule (at the highest and lowest concentrations). Among these statistical analyses, only the *t* tests that turn out to be significant are displayed in the figure (*<0.05, **<0.01, and ***< 0.001.). (C) Bar charts showing the fold change, which was derived from the ratio of the highest and the lowest GFP output for each system in each configuration.

Orthogonality (Figure 2B) was assessed by inducing each receiver separately for every AHL molecule. The results show that, while the rpa receiver is relatively promiscuous, responding to AHL molecules other than its cognate ligand, the other two systems (lux and cin) exhibit low cross-talk, which is minimal in the case of the latter.

### Performance of QS Systems at the Single-Strain Configuration

Both sender and receiver plasmids were cotransformed into a single strain. The sender modules were induced with 3-methylbenzoate (3MBz) to produce AHL signaling molecules. As shown in Figure 2A, sender activity successfully induced the receiver modules (cin, rpa, and lux) with outputs closely matching those observed when using synthetic AHLs. However, the basal GFP expression was higher than with synthetic AHL induction, leading to a lower fold change (Figure 2C). This may be due to leaky expression from the XylS-*Pm* promoter, which causes unintended AHL production and, consequently, GFP expression. Furthermore, the data show that even small amounts of 3MBz are sufficient to produce enough AHL to activate the receivers with the response quickly saturating. Notably, the cin system saturates at the lowest 3MBz concentration (0.047 mM), and GFP expression decreases at the highest levels of 3MBz.

### Performance of QS Systems at the Two-Strain Configuration

We characterized the systems in two separate strains to validate their performance at the consortia level. Senders and receivers were tagged with the fluorescent proteins eBFP and RFP, respectively, to facilitate tracking. The strains were mixed at a 1:1 ratio and cocultured for 5 h for the cin and rpa systems and 4 h for lux, based on their optimal performance. Using flow cytometry, we isolated the receiver population and measured GFP expression within this group (Figure 2A).

The results demonstrate that all three sender modules successfully induced the receiver modules when they were housed in separate cells. Unlike the configuration in which both modules were in the same strain, all three systems saturated at the lowest 3MBz concentration used. We hypothesize that this occurs because, while AHLs are still synthesized, the concentration that reaches the transcription factor in the receiver cells is much lower due to the diffusion of the molecules into the medium.

### Mathematical Model

We developed mathematical models to quantify the behavior of the three QS systems presented in this study and capture their differences numerically. Additionally, these models were conceived to support future efforts in designing more complex systems based on these three QS systems.

The behavior of the receiver modules is described using a Hill function by this equation:

1 $$\mathrm{GFP}=\beta +\frac{V_{m}{\left[\mathrm{AHL}\right]}^{n}}{K^{n}+{\left[\mathrm{AHL}\right]}^{n}}$$

where GFP represents GFP fluorescence as a function of the AHL concentration. The parameter β represents the baseline GFP expression in the absence of an AHL. *V*_m_ is the maximum fluorescence intensity representing the upper limit of GFP expression at saturation. *K* is the AHL concentration at which half-maximal activation occurs. Finally, *n* is the Hill coefficient, which determines the steepness of the response curve and reflects the level of cooperativity of the AHL binding to the transcription factor.

The QS systems with both sender and receiver plasmids, in either a single strain or two separate strains, are described by the sum of a Hill function and a decaying Hill-like function. The Hill function describes the sequential activations triggered by XylS activation by 3MBz, leading to AHL production and GFP expression through the action of LuxI, RpaI, CinI, LuxR, RpaR, and CinR. The decaying Hill-like function captures inhibitory effects observed at higher concentrations of 3MBz in some cases. The model is given by

2 $$y\left(x\right)=\frac{A_{1}x^{n_{1}}}{B_{1}+x^{n_{1}}}+\frac{A_{2}}{B_{2}+x^{n_{2}}}$$

where *y*(*x*) represents the logarithm of GFP fluorescence as a function of *x*, the 3MBz concentration. In the Hill function, $\frac{A_{1}x^{n_{1}}}{B_{1}+x^{n_{1}}}$ , the parameters *A*_1_, *B*_1_, and *n*_1_ represent the maximum activation level, the activation threshold, and the steepness of the activation curve, respectively. In the decaying Hill-like function, $\frac{A_{2}}{B_{2}+x^{n_{2}}}$ , the parameters *A*_2_, *B*_2_, and *n*_2_ correspond to the scaling factor, the decay threshold, and the steepness of the decay curve, respectively.

The values of the fitted parameters are shown in Table 1. For the receiver system, LuxR has the highest maximum fluorescence intensity (*V*_m_ = 809), while CinR shows the steepest activation curve (*n* = 8.64) and the lowest sensitivity to AHL (*K* = 1.04 × 10^–6^). For the one-strain system, lux and rpa systems have similar activation levels (*A*_1_ ≈ 12), while cin exhibits the steepest activation (*n*_1_ = 0.773) and the lowest threshold for activation (*B*_1_ = 0.0825). For the two-strain system, lux and cin have comparable activation levels (*A*_1_ = 4.4 and *A*_1_ = 8.7, respectively), while rpa shows the steepest decay (*n*_2_ = 5.1) and the highest threshold for decay (*B*_2_ = 15.7).

**Table 1:** Parameter Values for Receiver, 1-Strain, and 2-Strain Configurations

**receiver configuration parameters**				
system	β	*V*_*m*_	*K*	*n*
lux	13.5	809	2.73 × 10^–10^	1.64
rpa	386	634	2.5 × 10^–10^	0.97
cin	11.5	203	1.04 × 10^–6^	8.64

**1-strain** **configuration parameters**						
system	*A*_1_	*B*_1_	*n*_1_	*A*_2_	*B*_2_	*n*_2_
lux	12.3	1.7	0.253	4.57	1.25	0.878
rpa	12.2	1.67	0.58	4.0	0.65	1.03
cin	5.44	0.0825	0.773	0.141	0.0392	1.55

**2-strain** **configuration parameters**						
system	*A*_1_	*B*_1_	*n*_1_	*A*_2_	*B*_2_	*n*_2_
lux	4.4	0.0954	0.632	0.228	0.123	1.06
rpa	0.628	0.0369	0.751	49.2	15.7	5.1
cin	8.7	2.3	0.456	1.41	0.486	1.14

To further demonstrate how our model can inform design decisions, we performed a parameter sensitivity analysis (detailed in the SI, section: *Sensitivity analysis of the mathematical model*) to identify which parameters most strongly influence output in single- or two-strain setups.

## Concluding Summary

In this work, we have standardized and characterized three QS systems (rpa, cin, and lux) in the synthetic biology chassis P. putida KT2440. Although, P. putida KT2440 presents an orphan receptor from the LuxR family, Ppor^23^ and other P. putida strains encode different QS systems,^24–26^ none of these systems have been standardized or made available as a tool. With this work, we aim to contribute to improve modularization and interchangeability of quorum sensing devices using the SEVA standard. The wide applications of QS systems have been extensively described, ranging from coordinating gene expression across a microbial consortium^27^ and biomass valorization,^28^ to reducing the cost of expressing full metabolic pathways in the same strain (a strategy known as Division of Labor),^29^ as well as to understanding natural microbial communities^30^ and addressing scientific questions that cannot be solved through single-strain cultures.^13^ We propose that these standardized systems can now be leveraged to explore such scenarios in a broader range of bacterial species, offering alternatives that may outperform traditional model organism *E. coli*.

## Materials and Methods

### Strains, Media, and General Culture Conditions

The bacterial species used in this study were E. coli and P. putida; the specific strains are detailed in Supporting Table 1. Both were grown in Lysogeny Broth (LB) medium at 37 and 30 °C, respectively, with the corresponding antibiotic, and shaken at 200 rpm. M9 minimal media supplemented with 0.2% (v/w) citrate was only used during specific steps of the conjugation protocol.^31^ Antibiotics were used at the following concentrations: kanamycin (Km) 50 μg/mL and gentamicin (Gm) 10 μg/mL. The experiments were done in 100 mL flasks containing 25 mL of culture. Experiments performed in 96-well plates were inoculated using 100 μL of media at an initial OD600 = 0.1 and shaken at 400 rpm. Synthetic AHLs were purchased from Sigma-Aldrich (Saint Louis, Missouri), and were the following: N-Butyryl-DL-homoserine lactone (C4-HSL, 09945 Sigma-Aldrich), N-(β-Ketocaproyl)-l-homoserine lactone (3OC6-HSL, K3007 Sigma-Aldrich), N-(3-Hydroxytetradecanoyl)-DL-homoserine lactone (3OHC14-HSL, 51481 Sigma-Aldrich), and N-(p-Coumaroyl)-l-homoserine lactone (pC-HSL, 07077 Sigma). Inducer 3-methylbenzoate (3MBz; also known as m-toluic acid or m-toluate) was bought from Thermo Fisher Scientific (Waltham, Massachusetts) and prepared at a stock concentration of 500 mM (pH = 12).

### Sevarization of the Quorum Sensing Plasmids

All of the plasmids used during this work and their specifications are listed in Supporting Table 2. The SEVA plasmids chosen to standardize the systems were pSEVA621, pSEVA221, and pSEVA238 (SEVA Collection). The receiver modules were cloned from Kylilis and colleagues’ work,^18^ who kindly sent them to us. The sender modules were carried by plasmids bought on Addgene (for Addgene reference numbers, see Supporting Table 2). All of the constructs were assembled by classical restriction and ligation.^32^ QuikChange^33^ was used to eliminate the FseI restriction site on the plasmid pSEVA621_RpaCD, since this site is already present, flanking one of the modules of SEVA plasmids. Restriction enzymes, T4 DNA ligase, and Quick Ligation kit were from New England Biolabs (Ipswich, Massachusetts). DNA oligos (Supporting Table 3) were purchased from IDT (Coralville, Iowa), and Phusion polymerase was used for the amplifications for cloning (Thermo Fisher Scientific, Waltham, Massachusetts). Minipreps were performed using a Monarch Plasmid Miniprep kit (New England Biolabs, Ipswich, Massachusetts), and PCR products were purified with a Monarch PCR and DNA Cleanup kit (New England Biolabs, Ipswich, Massachusetts). Finally, the plasmids pTn7_J23102_BCD12_eBFP_rpoC and pTn7_J23102_BCD12_RFP_rpoC were constructed by the Golden Standard method (check Supporting Table 2 for intermediate Golden Standard plasmids)^34^ and then transferred to P. putida KT2440 through bacterial conjugation.^31^

### Induction of the Receivers with Synthetic AHL

Overnight cultures of transformed cells with the receivers’ plasmids were subjected to an automated workflow on the open-source liquid handler OT2 from Opentrons (Long Island City, New York). The first protocol was designed to create a 96-well plate with serial dilutions of the synthetic AHLs. The second consisted of diluting the cultures to a desired OD. The last protocol mixed the cells together with the different dilutions of the molecules. On the final 96-well plate, each well had a 100 μL volume of culture with an initial OD600 = 0.1. The plate was incubated at 30 °C while being shaken at 400 rpm. After 4 h, it was taken to a plate reader, where the turbidimetric (600 nm) and GFP fluorescence (ex: 485 nm/em: 512 nm) were measured. Two technical replicates and three biological replicates were performed for each condition. A control strain (P. putida KT2440 with the empty plasmid) was always added to the experiments to check that the measured fluorescence was a result of the AHL induction and not the intrinsic fluorescence of the strain. Supplementary Figure S2 compares the strain with the system and the control strain at both 0 and 10–5 M of the corresponding AHL induction.

### Induction of Receivers with the Senders

The two plasmids (corresponding to the sender and the receiver of each system) were either cotransformed in the same strain of P. putida or each of them was transformed in that host separately. For both characterizations, a similar automated workflow, as mentioned above, was used with two modifications: (i) the substitution of the inducer AHLs for 3MBz and (ii) in the case of separate senders and receivers, both strains were mixed in a 1:1 ratio after dilution of cultures to the corresponding OD before continuing to the next step. The plates were incubated at 30 °C and 400 rpm. Measurements were taken at 4 h for lux and 5 h for rpa and cin systems. Plates culturing cotransformed cells were measured in the plate reader as above. Plates with mixed sender and receiver cells were passed through a MACSQuant VYB Flow Cytometer (Miltenyi Biotec, Germany). The cultures were diluted with 150 μL of filtered LB to not surpass 20,000 events per second. Three biological replicates were performed for each condition. Several gates were applied to clean the data. A gate was first applied to the FSC-A and SSC-A density plots to select the population of bacteria and eliminate debris. The cytometer was set to pass 150,000 events that fitted into this gate. Then, doublets were eliminated from this selected population by applying a gate on the SSC-H versus SSC-A density plot. Finally, a gate was established to select the events presenting red fluorescence. The median values of the GFP-H channel (ex: 488 nm, em: 525/50 nm) of the selected events were plotted.

### Fitting of the Model

To fit the model to the experimental data in the 1-strain and 2-strain systems, we used the log-transformed GFP output. The log transformation was applied to account for the wide variation in GFP expression levels and to reduce the impact of large differences at higher expression values. By transforming the GFP output, the data were better able to capture trends across the entire range of 3MBz concentrations.

## Supplementary Material



## Notes

**Table S1:** Strains used in this work, **Table S2:** Plasmids used in this work, and **Table S3:** Oligos used in this work. **Figure S1:** Sensitivity analysis of the model and **Figure S2:** A comparison of the intrinsic autofluorescence of the P. putida KT2440 strain and the receiver strains (PDF)
