# Supplementary material for: Standardized Quorum Sensing Tools for Gram-Negative Bacteria
Source: ACS Synth Biol. 2025 Jun 6;14(6):2380–5. doi: 10.1021/acssynbio.5c00036 (PMC12186672; doi:10.1021/acssynbio.5c00036)
Supplement: Supplementary file 1 [file sb5c00036_si_001.pdf]

Supplementary material to Standardized Quorum Sensing Tools for Gram-Negative Bacteria

Paula Múgica-Galán,[†,‡] Jesús Miró-Bueno,[†] Ángeles Hueso-Gil,[†] Pablo Japón,[†] and Ángel Goñi-Moreno[*,†]

†*Systems Biology Department, Centro Nacional de Biotecnologia, CSIC, Darwin 3, 28049, Madrid, Spain*

‡*Universidad Politécnica de Madrid, Madrid, Spain*

E-mail: angel.goni@cnb.csic.es

Sensitivity analysis of the mathematical model

Along with fitting the data, we conducted a parameter sensitivity analysis in which each parameter $(A_1, B_1, n_1, A_2, B_2, n_2)$ was varied by $\pm 10\%$ around its fitted value, and we measured how the system's output metric responded (Fig. S1). Specifically, we computed:

$$S_i = \frac{\text{metric}_{\text{up}} - \text{metric}_{\text{down}}}{\text{baseline\_metric}},$$

where $\text{metric}_{\text{up}}$ and $\text{metric}_{\text{down}}$ are the model's outputs when each parameter is increased or decreased by 10%, respectively, and baseline_metric is the system's output with all parameters at their nominal (fitted) values. This measure S_i tells us the normalized range of change in the output metric for a $\pm 10\%$ swing in each parameter. For example, in the single-strain Lux system (Fig. S1A left), a $\pm 10\%$ perturbation to A_1 yields an S_i of approximately 0.82,

meaning the difference in output between $(A_1 + 10\%)$ and $(A_1 - 10\%)$ is about 82% of the baseline value. Such a high S_i indicates that tuning A_1 can substantially shift the circuit's expression level—an immediately testable prediction. This could be verified experimentally by adjusting the promoter or ribosome binding site controlling A_1's gene and measuring the new expression range. Similarly, other parameters with large positive or negative S_i-values stand out as key levers for circuit optimization: small changes will significantly affect overall performance. For instance, the single-strain Cin system shows a high sensitivity to A_1 as well (Fig. S1A right), while Rpa stands out for its sensitivity to A_2 (Fig. S1A middle). These insights directly guide engineering by identifying which regulatory elements to modify first.

Table S1: Strains used in this work.

Strain	Description	Reference
E. coli pir2	F- δ(argF-lac)169 rpoS(Am) robA1 creC510 hsdR514 endA recA1 uidA(δMluI)::pir+	Invitrogen
E. coli TOP10	F- mcrA Δ(mrr-hsdRMS-mcrBC) Φ80lacZΔM15 ΔlacX74 recA1 araD139 Δ(araleu)7697 galU galK rpsL (StrR) endA1 nupG	Invitrogen
E. coli DH10B	F– mcrA Δ(mrr-hsdRMS-mcrBC) Φ80lacZΔM15 ΔlacX74 recA1 endA1 araD139 Δ(ara-leu)7697 galU galK λ– rpsL(StrR) nupG	Invitrogen
P. putida KT2440	Prototrophic, wild-type strain derived of *P. putida* mt-2 without pWW0 plasmid	[1]
P. putida KT2440 RFP	*P. putida* KT2440 with RFP inserted at the attTn7 site, GmR	This work
P. putida KT2440 eBFP	*P. putida* KT2440 with eBFP inserted at the attTn7 site, GmR	This work

Table S2: Plasmids used in this work.

Plasmid	Description	Reference
lux device composite	pSB1C3 vector, CamR, pMB1 origin, LuxR, pLux, GFPmut3b	[2]
rpa device composite	pSB1C3 vector, CamR, pMB1 origin, RpaR, pRpa, GFPmut3b	[2]
cin device composite	pSB1C3 vector, CamR, pMB1 origin, CinR, pCin, GFPmut3b	[2]
pTD103LuxI_sfGFP	pZ vector, KmR, LuxI, LuxR, sfGFP	Addgene plasmid nº 48885[3]
Bsrs103-RpaR-RpaI	pTD103 vector, KmR, RpaR, RpaI	Addgene plasmid nº 85159[4]
C332	pSB1C3 vector, CamR, CinR, pCin, GFP	Addgene plasmid nº 141124[3]
pSEVA621_LuxR	pSEVA621 derived, GmR, RK2 origin, luxR, pLux→ GFPmut3b	This work
pSEVA621_RpaR	pSEVA621 derived, GmR, RK2 origin, rpaR, pRpa→ GFPmut3b	This work
pSEVA621_CinR	pSEVA621 derived, GmR, RK2 origin, cinR, pCin→ GFPmut3b	This work
pSEVA238_LuxI	pSEVA238 derived, KmR, pBRR1 origin, XylSPm→ LuxI	This work
pSEVA238_RpaI	pSEVA238 derived, KmR, pBRR1 origin, XylSPm→ RpaI	This work
pSEVA238_CinI	pSEVA238 derived, KmR, pBRR1 origin, XylSPm→ CinI	This work
pSEVA221_LuxR	pSEVA221 derived, KmR, RK2 origin, LuxR, pLux→ GFPmut3b	This work
pSEVA221_RpaR	pSEVA221 derived, KmR, RK2 origin, RpaR, pRpa→ GFP-mut3b	This work
pSEVA221_CinR	pSEVA221 derived, KmR, RK2 origin, CinR, pCin→ GFPmut3b	This work
pTn7-19[g1R]	pTn7 derived, compatible with Golden Standard (BsaI sites, 2A and I1 fusion sites), lacZ fragment.	Alejaldre, L. (Unpublished)
pTn7_J23102_BCD12_eBFP_rpoC	pTn7-19[g1R] vector, GmR, pJ23102→ eBFP	This work
pTn7_J23102_BCD12_RFP_rpoC	pTn7-19[g1R] vector, GmR, pJ23102→ RFP	This work

Table S3: Oligos used in this work.

**Primer name**	**Sequence**
CinI_FWD	GAAATCGTAGCCCGGGGGGGGAGTGTTACGAGTATGTTCG
CinI_RV	CTGTTAGCATAAGCTTTGCTGCCATCTCCAGGAATTGG
RpaI_FWD	TCGTAATGGCCCCGGGAAAGAGGAGAAAGGTACCATGC
RpaI_RV	ACGTTGATACAAGCTTTCATGAAATCACCTGGAATTCC
LuxI_FWD	AGGTCCATATCCCGGGAAAGAGGAGAAAGGTACCATGAC
LuxI_RV	CTGGAATGCAAAGCTTTTACGCTGCAAGGGCGTAATTTTCG
QSecoRI_FWD	CTTAATCGTGGAATTCTTTACAGCTAGCTCAGTCCTAGG
QSkpnI_FWD	CTTAGTAGATGGTACCTTTACAGCTAGCTCAGTCCTAGG
QSxbaI_FWD	GCTGAATCGTTCTAGATTTACAGCTAGCTCAGTCCTAGG
QShindII_RV	CAATGTCCTAAAGCTTTTATTATTTGTATAGTTCATCCATGCC
QSbamHI_RV	CTAAGTCTGAGGATCCTTATTATTTGTATAGTTCATCCATGCC
Rpa_Qc_FWD	CTACATCATGGCGGGCCTGCCGTCGCGCAATGCCGGACTAC
Rpa_Qc_RV	CAGGCCCGCCATGATGTAGGCGGTAAATCCGCAGCTCGCG

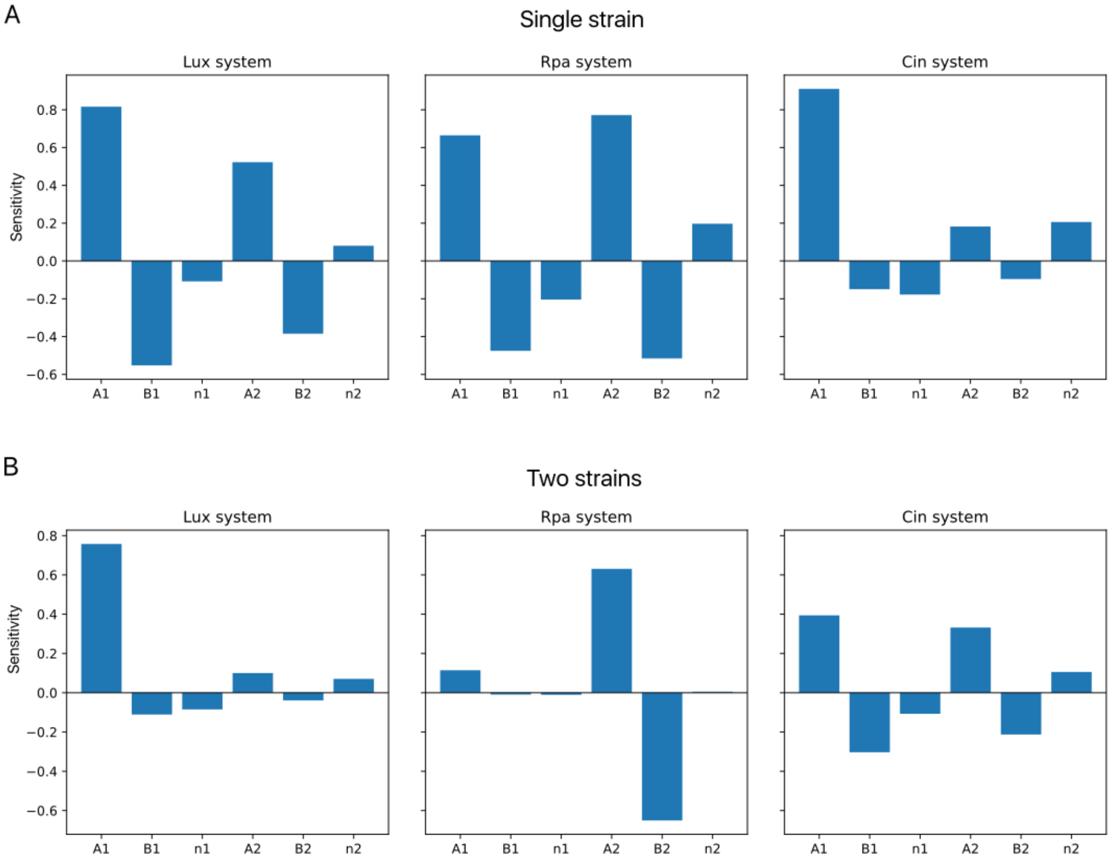

Figure S1: Sensitivity analysis of the mathematical model. A) For single-strain constructs, Cin exhibits the largest positive sensitivity to A_1, indicating that increasing this parameter most effectively boosts expression in that system. Rpa shows a higher sensitivity to A_2, while Lux has high positive value for A_1. Notably, all three exhibit negative sensitivities to B_1 and B_2, meaning increases in these parameters reduce overall output. B) In the two-strain setup, Lux still depends strongly on A_1, albeit with a slightly smaller magnitude, while Rpa remains most sensitive to A_2. Cin retains a notable positive dependence on A_1 and A_2. Together, these findings identify which parameters are prime targets for upregulating expression (large positive S_i) and which may be tuned to limit maximum output (large negative S_i).

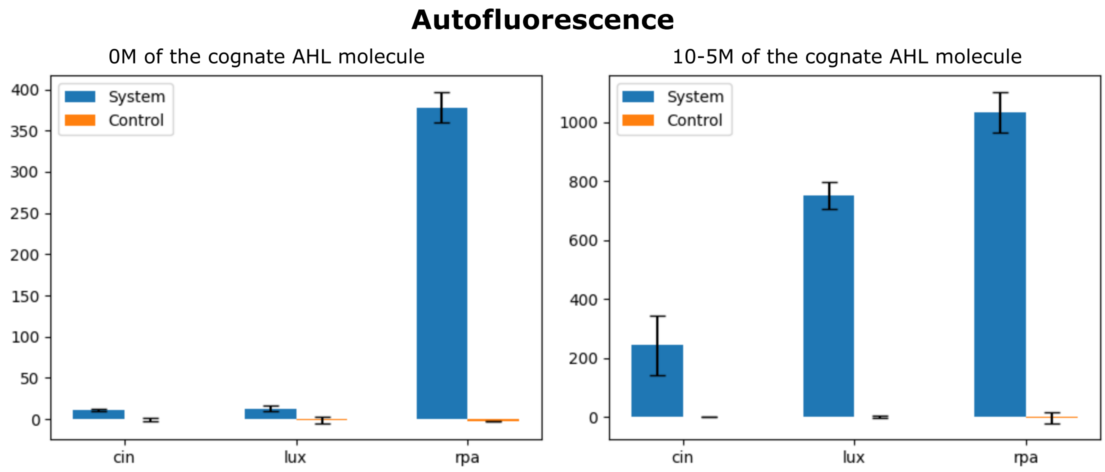

Figure S2: Comparison of the instrinsic autofluorescence of the *P. putida* KT2440 strain and the receiver strains. Fluorescence levels were compared at two concentrations: 0M and 10-5M of AHL. The graph shows that the increase of fluorescence as a response to the presence of the AHL inducer does not come from the empty strain.

References

(1) Nelson, K.; Weinel, C.; Paulsen, I.; Dodson, R.; Hilbert, H.; Martins dos Santos, V.; Fouts, D.; Gill, S.; Pop, M.; Holmes, M.; others Complete genome sequence and comparative analysis of the metabolically versatile Pseudomonas putida KT2440. *Environmental Microbiology* **2003**, *5*, 630–630.

(2) Kylilis, N.; Tuza, Z. A.; Stan, G.-B.; Polizzi, K. M. Tools for engineering coordinated system behaviour in synthetic microbial consortia. *Nature communications* **2018**, *9*, 2677.

(3) Alnahhas, R. N.; Sadeghpour, M.; Chen, Y.; Frey, A. A.; Ott, W.; Josić, K.; Bennett, M. R. Majority sensing in synthetic microbial consortia. *Nature Communications* **2020**, *11*, 3659.

(4) Scott, S. R.; Hasty, J. Quorum sensing communication modules for microbial consortia. *ACS synthetic biology* **2016**, *5*, 969–977.
